# Yeast quiescence exit swiftness is influenced by cell volume and chronological age

**DOI:** 10.15698/mic2018.02.615

**Published:** 2017-12-06

**Authors:** Damien Laporte, Laure Jimenez, Laëtitia Gouleme, Isabelle Sagot

**Affiliations:** 1 CNRS, Université de Bordeaux - Institut de Biochimie et Génétique Cellulaires, UMR5095 - 33077 Bordeaux cedex, France.

**Keywords:** S. cerevisiae, quiescence, aging, cell size

## Abstract

Quiescence exit swiftness is crucial not only for micro-organisms in competition for an environmental niche, such as yeast, but also for the maintenance of tissue homeostasis in multicellular species. Here we explore the effect of replicative and chronological age on *Saccharomyces cerevisiae* quiescence exit efficiency. Our study reveals that this step strongly relies on the cell volume in quiescence but is not influenced by cell replicative age, at least for cells that have undergone less than 10 divisions. Furthermore, we establish that chronological age strongly impinges on cell’s capacities to exit quiescence. This effect is not related to cell volume or due to cell’s inability to metabolize external glucose but rather seems to depend on intracellular trehalose concentration. Overall, our data illustrate that the quiescent state is a continuum evolving with time, early and deep quiescence being distinguishable by the cell’s proficiency to re-enter the proliferation cycle.

## INTRODUCTION

Most cells spend the majority of their life in a non-dividing state. A non-dividing cell is considered as senescent when it is still metabolically active, but will never re-enter proliferation. By opposition, quiescent cells cease to proliferate only temporarily and will divide again in response to external clues. These definitions are operational and with time, quiescent cells may lose their abilities to give rise to a progeny and enter senescence or eventually die. This loss of re-proliferation capacities is at the heart of the cellular aging process 1,2,3,4.

*Saccharomyces cerevisiae* has been an instrumental model for studying cellular aging 5,6,7,8. In this organism, as in all asymmetrically dividing eukaryotes, two aging paradigms have been defined. The replicative age is the number of divisions a cell can potentially undergo before entering senescence 9,10. As such, a yeast mother cell can produce a limited number of daughter cells, typically from 20 to 45, depending on the experimental conditions and the genetic background. The chronological age is defined as the time a non-dividing cell can stay alive 11,12,13. During both the replicative and the chronological aging processes, the accumulation of damaged macromolecules until a threshold is supposed to lead to senescence 14,15. Interestingly, in budding yeast, chronological age reduces cell’s replicative capacity, as cells that have been quiescent for a long time have a shortened replicative lifespan 16,17. Conversely, it has been proposed that replicative age influences cell’s ability to maintain quiescence, since daughter cells have been described to have a better survival prognostic in quiescence than mother cells 18, but this remains controversial 19,20,21.

A vast amount of environmental cues 22 and a large panel of genes have been shown to impact *S. cerevisiae* cell survival in quiescence 23, yet most of these studies do not distinguish defects in quiescence establishment, maintenance or exit. Recent data support the idea that quiescence exit in *S. cerevisiae* is temporally organized and controlled by distinct sets of genes, including *XBP1*, *SRL3*, *WHI5*, *SSD1*, *LSM1*, *MPT5* and *MSA1/2* 24,25,26. However, to date, almost nothing is known about cellular properties that influence quiescence exit swiftness, a step that is crucial, not only for micro-organisms in competition for an environmental niche, such as yeast, but also for an efficient maintenance of tissue homeostasis in multicellular species.

Here we explore the influence of replicative and chronological age on yeast quiescence exit swiftness. We show that this step strongly relies on the cell volume in quiescence. Our data also reveal that this process is not influenced by the cell replicative age, at least for cells that have undergone less than 10 divisions. Furthermore, we establish that chronological age strongly impinges on quiescence exit efficiency and provide evidences that this effect is not related to cell volume or due to cell’s inability to metabolize external glucose but most probably depends on the trehalose intracellular reservoir.

## RESULTS AND DISCUSSION

### Quiescence efficiency, cell volume and replicative age

**Figure 1:**
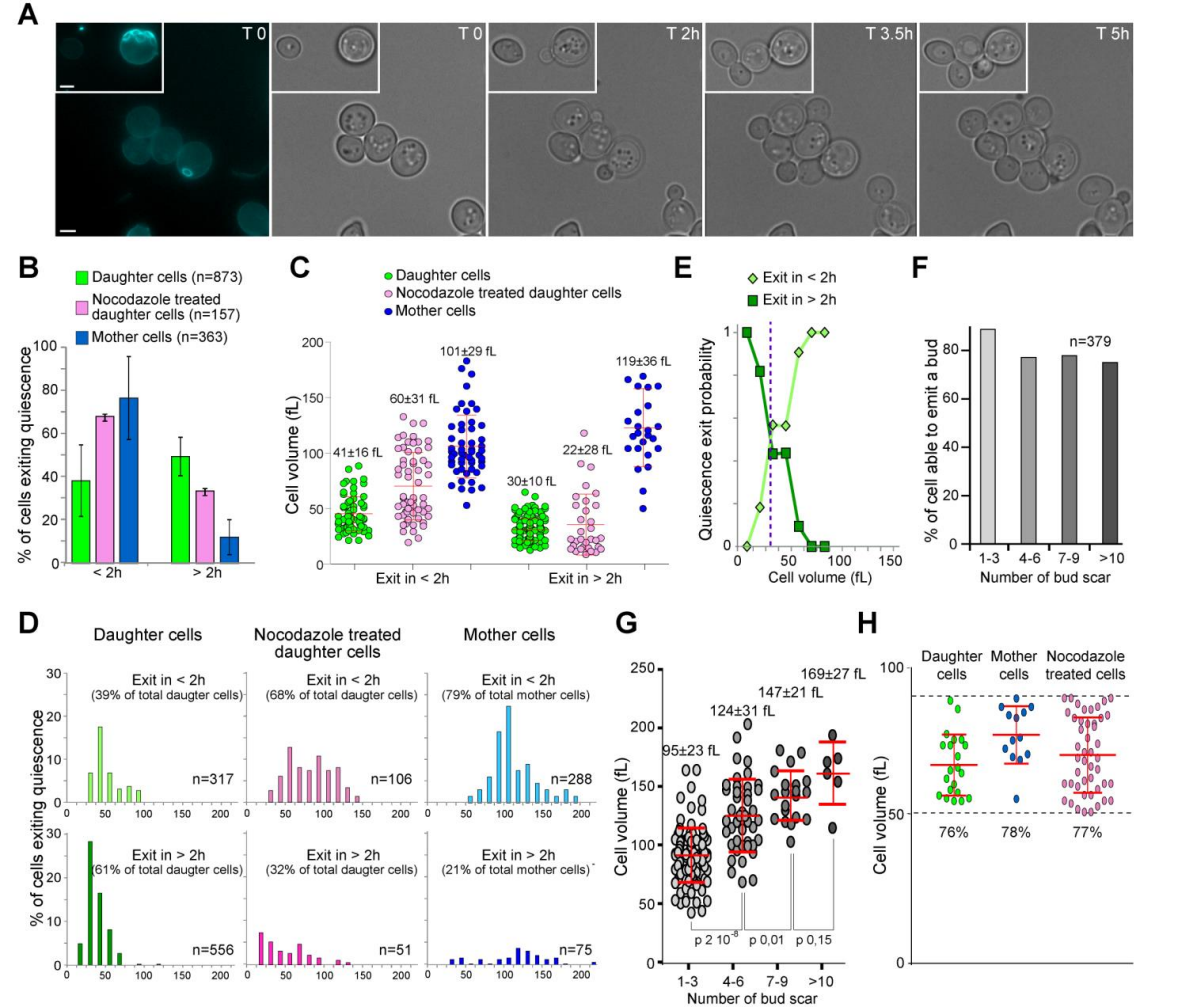
FIGURE 1: Daughter and mother cell quiescence exit efficiency. **(A)** Image series of 7-day-old wild type mother and daughter cells exiting quiescence on a YPD-containing microscope agarose pad. The first image shows a calcofluor white staining revealing bud scars (blue). Bar is 2 µm. **(B) **Percentage of 7-day-old wild type mother and daughter cells able to exit quiescence in less than 2 h (< 2 h) or between 2 and 6 h (> 2 h) on a YPD-containing microscope agarose pad at room temperature (N=3, the number of cell scored is indicated). Histograms are means and bars are SD. **(C)** Initial volume in function of the time needed for bud emergence. Cells are the same as in (B). Median cell volume and SD are indicated. **(D)** Volume distribution according to quiescence exit swiftness. Cells are the same as in (B), top graphs: emit a bud in less than 2 h; lower graphs: emit a bud within 2 to 6 h. The percentage of each sub-populations and the number of analyzed cells are indicated. **(E) **Probability of a daughter cell to exit quiescence in less or more than 2 h, according to its volume in quiescence. **(F)** Percentage and **(G) **volume of mother cells able to exit quiescence in less than 2 h, accordingly to their replicative age. Median cell volume and SD are indicated. **(H) **Cell ability to exit quiescence in less than 2 h within an identical cell volume range (50 to 90 fL). In all panels, mother cells are in blue, daughter cells in green, and nocodazole treated cells in pink.

To get an insight into the influence of cell replicative age on quiescence exit efficiency, wild type cells were grown in liquid YPDA medium at 30°C. After 7 days of culture, cells were stained with calcofluor white, a dye that reveals bud scars and allows to distinguish daughter cells (zero scar) from mother cells (the number of scars reflecting the number of divisions). After staining, quiescence exit was triggered by re-feeding the cells onto a YPD-containing-microscope agarose pad. Cells were tracked individually and imaged every hour, from the time they were deposited onto the microscope pad up to 6 h, a time after which extensive cell proliferation prevented us to undoubtedly track each cell (Fig. 1A). A cell was considered as exiting quiescence when it emitted a bud.

We have followed 873 daughter cells and 363 unbudded mother cells that were capable of exiting quiescence within 6 h. As shown in Fig. 1B, ≈ 80% of the mother cells re-entered the proliferation cycle in less than 2 h. By contrast, only ≈ 40% of daughter cells were able to exit quiescence within the same time frame. Thus, mother cells exit quiescence more rapidly than daughter cells.

When yeast cells are actively proliferating, bud emergence takes place only after a critical cell size is reached. This size control occurs in the G1 phase of the cell cycle. Consequently, smaller cells have a prolonged G1 duration. *S. cerevisiae* divides asymmetrically and gives rise to daughter cells that are smaller than their mothers 27,28,29. We therefore hypothesized that mother cells were re-entering the proliferation cycle faster than daughter cells simply because they were larger than daughter cells. As such, mother cells would need less time to reach a critical size required for bud emergence upon quiescence exit. To test this idea, we primarily measured the quiescence exit critical volume i.e. the median volume at which 7 days old daughter cells were emitting a bud after re-feeding on a YPD-containing microscope agarose pad, irrespectively of the time spent on the pad, and found 58 +/- 12 fL (Fig. S1A). Then, we measured cell’s initial volume in quiescence i.e. just after cell deposition onto the YPD-containing microscope agarose pad. As shown in Fig. 1C and D, mother cells that exited quiescence in less than 2 h displayed an initial median cell volume of 101 +/- 29 fL, well above the quiescence exit critical volume. Daughter cells that exited quiescence in less than 2 h had an initial median cell volume of 41 +/- 16 fL, a volume close to the quiescence exit critical volume (p-value 0.02). By contrast, daughter cells that exited quiescence in more than 2 h were meaningfully smaller (30 +/- 10 fL), and thus, markedly below the quiescence exit critical volume (p-value 1.10^-35^, Fig. 1C-D). This suggests that daughter cell quiescence exit efficiency is primarily influenced by the cell volume in quiescence.

To verify this hypothesis, we have tried to find a way to increase artificially daughter cell volume in quiescence in a wild type population, since mutations that are known to influence cell volume may also interfere with quiescence survival and exit properties. We used nocodazole, a drug that depolymerizes microtubules and causes a cell cycle arrest in metaphase without inhibiting cell growth 30. Proliferating cells were treated with nocodazole as described in the materials and methods section. After 7 days, we measured that daughter cells treated with nocodazole were significantly larger than untreated daughter cells (median cell volume of 53 +/- 34 fL and 33 +/- 14 fL respectively, p-value 1.10^-22^). Interestingly, among daughter cells treated with nocodazole, ≈ 70% were able to exit quiescence in less than 2 h compare to ≈ 40% for the untreated daughter cell population (Fig. 1B). Accordingly, the nocodazole treated population of daughter cells exiting quiescence in less than 2 h had an initial median cell volume of 60 +/- 31 fL (Fig. 1C-D), a volume similar to the quiescence exit critical volume (58 +/- 12 fL, Fig. S1A). From those experiments, we concluded that the initial cell volume is critical for daughter cell quiescence efficiency.

The cell division cycle can be viewed as controlled by sizers and timers. Sizers involve that cells pass a volume threshold, while timers require that cells wait a fixed amount of time, independently of their volume 31. In proliferating yeast, daughter cells show a strong sizer control 32,33. Similarly, the above data indicate that daughter cell quiescence exit efficiency is mostly controlled by a sizer. Using the data of Fig. 1C-D, we plotted the distribution of the cell initial volume in function of the time needed for quiescence exit and found that statistically, daughter cells below the volume threshold of 35 fL have a high probability to exit quiescence in more than 2 h (Fig. 1E).

In contrast to daughter cells, in proliferating yeast, G1 duration in mother cells was shown to be essentially independent of the cell volume and mostly controlled by a timer 32,33. Similarly, the cell volume distribution observed in Fig. 1C-D seems to indicate that the volume in quiescence did not influence mother cell quiescence exit efficiency. In fact, mother cells that exited quiescence in more than 2 h were initially slightly larger (119 +/- 36 fL) than mother cells that exited quiescence in less than 2 h (101 +/- 29 fL, p-value = 0.04). Therefore, a sizer cannot account for the variation in mother cell quiescence exit efficiency. As the cell volume is known to increase with cell replicative age 9,34, we envisioned that mother cell replicative age could negatively influence quiescence exit swiftness. We thus scored the percentage of mother cells that were capable of exiting quiescence in less than 2 h in function of replicative age. Several replicative age categories were distinguished: 1-3, 4-6, 7-9 and more than 10 bud scars. As expected, the more bud scar a mother cell displayed, the less frequent it was in the mother cell population (Fig. S1B). In fact, the proportion of cells with n buds scar is theoretically close to 1/2^n+1^. Thus, cells with more than 10 bud scars were rarely observed using our individual cell approach and could not be analysed with a good statistical significance. Importantly, Fig. 1F showed that ≈ 80% of mother cells were able to exit quiescence in less than 2 h whatever their replicative age, at least for cells with less than 10 bud scars. Besides, as expected, within cells capable of exiting quiescence in less than 2 h, the mother cell volume increased with replicative age (Fig. 1G). Thus, mother cell quiescence exit efficiency is neither influenced by the cell volume nor by the cell replicative age, but is rather mostly controlled by a “timer”, at least for cells that have undergone less than 10 divisions. The molecular nature of this timer remained to be identified, but may involve a difference in the amount of nuclear Whi5, a Rb homolog that inhibits start in G1 35.

Finally, we compared quiescence exit swiftness of daughter and mother cells within the same volume range (50 to 90 fL) and observed that the majority of the cells (≈ 80%) exited quiescence in less than 2 h whatever their replicative age (Fig. 1H). Therefore, when daughter and mother cells have a similar initial volume, they re-enter the proliferation cycle with the same efficiency. All together our data demonstrate that replicative age per se has no influence on quiescence exit swiftness, at least for mother cells that have undergone less than 10 divisions.

### Chronological age strongly influences quiescence exit efficiency

**Figure 2:**
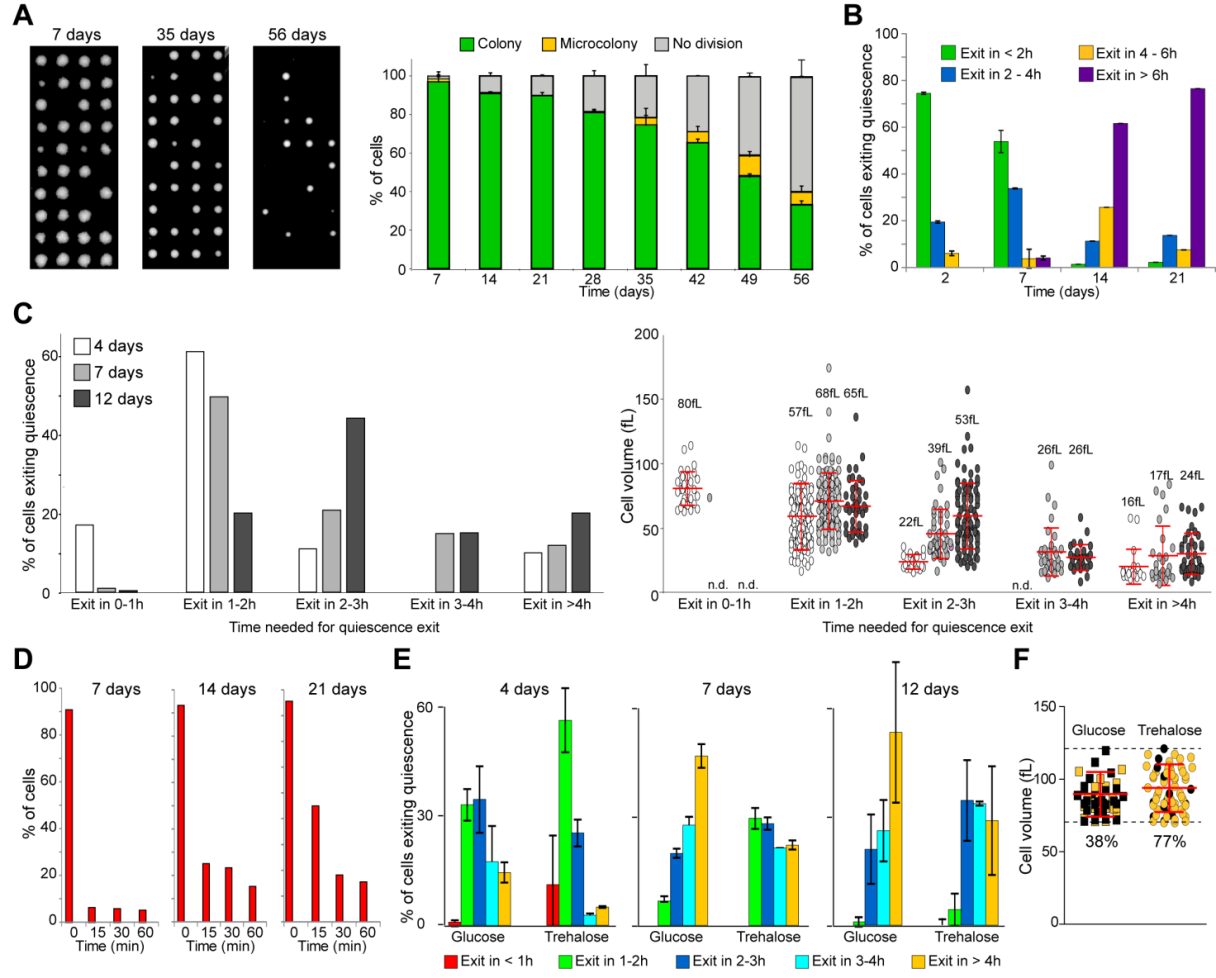
FIGURE 2: Quiescence exit swiftness decreases with chronological age. **(A)** Cell ability to form a colony decreases with chronological age. Percentage of wild type prototroph FY4 cells able to form a colony in function of the time spend in YPDA at 30°C. Individual cells were isolated by micro-manipulation and allowed to grow 3 days at 30°C on YPDA plates (examples are shown). The percentage of cells able to give rise to a colony or a micro-colony (less than 10 divisions) was scored (n=240, N=3). **(B)** Quiescence exit efficiency in function of chronological age. Wild type cells were grown in YPDA at 30°C for the indicated time and re-fed on a YPD-containing microscope agarose pad. Quiescence exit efficiency was scored for each time point (n>300, N=2). **(C)** Quiescence exit efficiency in function of chronological age. Wild type cells were grown in YPDA at 30°C for the indicated time, then re-fed on a YPD-containing microscope agarose pad. Left panel: percentage of cells able to exit quiescence within the indicated time frame. Right panel: cell initial volume in quiescence; median volume and SD are indicated. **(D)** Actin body mobilization upon quiescence exit. Wild type cells expressing the actin binding protein Abp1-3xGFP were grown in YPDA at 30°C. Glucose (4% final) was added to the medium and the actin cytoskeleton organization was analyzed (n>200, N=2). **(E)** Trehalose influences quiescence exit efficiency. Wild type prototroph CEN-PK cells were grown 24 h in YP pH 5 containing 4% glucose or 2% glucose + 2% trehalose, then washed and inoculated in YP without sugar for the indicated time. Quiescence exit was triggered on a YPD-containing microscope agarose pad, for each time point n>100 N=2. **(F)** Cell’s ability to exit quiescence after 12 days of culture in the presence of 4% glucose or 2% glucose + 2% trehalose, within the initial volume range of 70 to 120 fL (n>45). Number are the percentage of cells exiting quiescence in less than 2 h (in yellow) within all the cells capable of exiting quiescence (quiescence exit in > 2 h in black). Histograms represent means and bars are SD.

We then wondered what was the influence of the time spent in quiescence, i.e. chronological age, on quiescence survival. Wild type cells were grown in YPDA at 30°C. A small volume of the culture was then transferred onto a YPDA plate and individual cells were separated by micro-manipulation 36. After 3 days, colonies were scored. As shown in Fig. 2A, the number of cells that were able to give rise to a progeny decreased with the culture age. After 8 weeks, less than 40% of the cells were able to re-enter the cell cycle and form a colony. This clearly demonstrates that the time spent in a non-proliferative state strongly influences quiescent cell long-term survival. It remains to be determined if, with time, quiescent cells die or just lose their capacities to proliferate and become senescent. The molecular pathways that account for the loss of reproducing capacity with chronological age are still poorly understood, but several studies point to mitochondria as a key organelle 37,38.

During the first 3 weeks, the number of cells able to exit quiescence remained nearly constant (≈ 90%, Fig. 2A). Therefore, within this time window, we could address the influence of the chronological age on quiescence exit efficiency, independently of cell survival capacities. Cells cultured for 2, 7, 14 and 21 days were re-fed onto a YPD-containing microscope agarose pad and individual cell quiescence exit efficiency was monitored as previously. We observed that more than 90% of 2 or 7-days old cells were able to exit quiescence in less than 4 h. By contrast, the majority of the 14 or 21-days old cells needed more than 6 h to re-bud (Fig. 2B). This indicates that chronological age strongly influences quiescence exit swiftness. To get a more detailed view of the chronological age effect on quiescence exit efficiency, we have repeated the experiment with a better temporal resolution and measured the cell volume before quiescence exit. First, the initial median cell volume did not significantly vary within the first 12 days (Fig. S1C). Second, as expected from Fig. 2B, the older the cell, the longer was the time needed to exit quiescence (Fig. 2C, left panel). Third, for a given culture age, the larger the cell, the faster quiescence exit was, as expected from Fig. 1. Finally and interestingly, this experiment revealed that the quiescence exit delay observed for chronological aged cells was not due to a difference in cell volume in quiescence (Fig. 2C, right panel). Indeed, chronologically old cells that are delayed for quiescence exit were not smaller than their younger counterpart. This suggests that, with chronological age, quiescence exit efficiency is increasingly influenced by a timer.

To get an insight into the molecular mechanism underlying this "timer" effect, we tested the possibility that the observed delay in quiescence exit was due to a slowdown of glucose metabolization upon refeeding. We took advantage of actin bodies, stable actin structures specifically assembled in non-proliferating cells. In 7 days old cells, actin bodies are mobilized within seconds upon glucose addition, and concomitantly depolarized actin patches and cables are reassembled 36,39. These processes strictly depend on glucose utilization by the glycolytic pathway 36. Wild type cells expressing the actin binding protein Abp1 fused to 3xGFP were grown in YPDA at 30°C for 7, 14 and 21 days. As expected, before re-feeding, more than 90% of the cells displayed actin bodies whatever the cell chronological age (Fig. 2D). As previously shown 39, 15 min after glucose addition, actin bodies were fully disassembled in more than 90% of the 7 days old cells (Fig. 2D). Intriguingly, actin body mobilization was slightly delayed after 14 and 21 days of culture, as 25% and 45% of the cells still exhibit actin bodies 15 min after glucose addition, respectively. Yet, after 30 min, actin bodies were fully disassembled in more than 80% of the cells whatever the culture age (Fig. 2D). Thus, in chronologically aged cells, even if there was a delay in actin body mobilization that could be due to a slowdown of glucose metabolization, it could not fully account for the observed > 4 h quiescence exit postponement.

We then envisioned that the timer influencing chronologically aged cell quiescence exit swiftness could rely on intracellular trehalose stockpile. Trehalose is known to accumulate in cells undergoing carbon source limitation and this disaccharide can account for as much as 20% of the cell dry weight 20,40,41,42,43,44. Trehalose is essential for yeast long-term survival 45,46,47 and is involved in quiescence exit 20,48,49,50. If the trehalose intracellular content is one of the key for quiescence exit efficiency as cells age, artificially increasing the trehalose stockpile would shorten the observed delay in chronologically old cell quiescence exit. To increase intracellular trehalose content, we utilized a prototroph CEN-PK strain that can uptake and accumulate this disaccharide at least 20 times more when grown in the presence of glucose and trehalose than in the sole presence of glucose 51. We therefore analysed quiescence exit swiftness of CEN-PK cells grown in the presence of glucose or glucose plus trehalose. To exclude possible dissimilarities in quiescence survival, only cells able to exit quiescence within 6 h were compared. We found that, whatever the chronological age, cells grown in the presence of trehalose exited quiescence faster than cells fed only with glucose (Fig. 2E and S1D). This effect was not influenced by the cell initial volume since within the same volume range (70 to 120 fL), 77% of the 12 days old cells grown in the presence of both glucose and trehalose were capable to exit quiescence in 2 h while only 38% of the cells grown in the presence of glucose alone were able to do so. This indicates that trehalose intracellular concentration influenced quiescence exit swiftness independently of the cell volume in quiescence. As trehalose intracellular stockpiles are consumed during chronological age 21,40,50, we speculate that the decrease in trehalose reservoir may account for the observed decline in quiescence exit swiftness with age.

Overall, our study reinforces the idea that quiescence is not a uniform cellular state 52. It was elegantly shown by Broach and colleagues that yeast cells can enter distinct quiescent states depending on the environmental cues that have triggered quiescence establishment 53. Here we show that chronological age strongly influences not only quiescent cell survival but also the cell’s ability to exit quiescence efficiently. Consequently, at a population scale, quiescence is a continuum evolving with time, as it is possible to distinguish early quiescence from deep quiescence based on cell’s ability to re-enter the proliferation cycle. This findings echoes what has been found in other yeast species 54,55 and in some multicellular models 56,57.

## MATERIALS AND METHODS

### Yeast strains and growth conditions

All the strains used in this study are, unless specified, isogenic to BY4741 available from GE Healthcare Dharmacon Inc. The strain expressing ABP1-3xGFP was described in 39. For Fig. 2A we utilized a FY4 prototroph strain. For Fig. 2E-F and S1D, we utilized a prototroph CEN.PK113-7D strain (a gift from J-L Parrou 51).

Cells were grown in YPDA at 30°C in flasks as described previously 39. For the nocodazole experiment, proliferating cells (OD 2-4) were incubated 6 h with 15 µg/ml nocodazole (M1404 - Sigma), washed twice in YPA, and inoculated at the original density in YPA. For Fig. 2E and F, cells were pre-cultured 24 h in liquid YPDA, transferred in liquid YPA pH 5 containing either 4% glucose or 2% glucose + 2% trehalose. After 24 h, cells were washed twice in YPA pH 5 and inoculated at the original density in YPA pH 5. A similar protocol was used for Fig. S1D, but cells were left in YPA containing either 4% glucose or 2% glucose + 2% trehalose.

### Cell staining, viability and quiescence exit

For quiescence exit assay, cells grown for the indicated time in liquid YPDA were incubated 2-3 min in YPD and spread onto a 2% agarose pad containing YPD. For Fig. 2E and F, cells grown in the presence of trehalose were stained with concanavaninA-FITC (0.2 mg/mL, Sigma-Aldrich, Saint Louis, MI, USA, as described in 58), then mixed with unstained cells grown in the presence of glucose, and both were imaged simultaneously on the same YPD-containing microscope agarose pad. This allow us to compared quiescence exit efficiency of two cell populations in the exact same quiescence exit experimental conditions.

To identify mother and daughter cells, cells were incubated 5 min with Calcofluor white (20 µg/mL, Sigma-Aldrich, Saint Louis, MI, USA) and washed once with YPD.

Colony forming capacity was addressed by micro-manipulation of individual cells on YPDA plates as described in 36. Plates were then incubated 3 days at 30°C.

For glucose sensing experiment (Fig. 2D), cells were grown in YPDA at 30°C for the indicated time, then 4% glucose (final) was added into the old medium.

### Fluorescence Microscopy

Cells were observed in a fully automated Zeiss 200M inverted microscope (Carl Zeiss, Thornwood, NY, USA) equipped with a MS-2000 stage (Applied Scientific Instrumentation, Eugene, OR, USA), a Lambda LS 300 W xenon light source (Sutter, Novato, CA, USA), a 100X 1.4NA Plan-Apochromat objective, and a 5 position filter turret. All the filters are from Chroma Technology Corp. Images were acquired using a CoolSnap HQ camera (Roper Scientific, Tucson, AZ, USA). The microscope, camera, and shutters (Uniblitz, Rochester, NY, USA) were controlled by SlideBook software 5.0. (Intelligent Imaging Innovations, Denver, CO, USA). Image analysis were done with Slidebook 5.0 or Image J.

### Statistical analysis

Cell size measurements were done using the spheroid formula: 4/3*π*a^2^*c, where a and c are the equatorial and polar radius respectively.

All the statistical analysis were done using GraphPad Prism 5 (GraphPad Software, Inc. La Jolla, USA) or Excel (Microsoft).

Quiescence daughter cell exit probability (Fig. 1E) was determined as follow: NV_x-y<2h_ is the cell numbers (N) scored for the volume interval (Vx-y) that exit quiescence in less than 2 h. NV_x-y>2h_ is the cell numbers (N) scored for the volume interval (Vx-y) that exit quiescence in more than 2 h. The probability to exit quiescence within less than 2 h is thus P_<2h_ = NV_x-y<2h_ / (NV_x-y<2h _+ NV_x-y>2h_) and the probability to exit quiescence in more than 2 h is P_>2h_ = 1- P_<2h_.

## SUPPLEMENTAL MATERIAL

Click here for supplemental data file.

All supplemental data for this article are also available online at http://microbialcell.com/researcharticles/yeast-quiescence-exit-swiftness-is-influenced-by-cell-volume-and-chronological-age/.
