# Supplementary material for: Yeast quiescence exit swiftness is influenced by cell volume and chronological age
Source: Microb Cell. 2017 Dec 6;5(2):104–11. doi: 10.15698/mic2018.02.615 (PMC5798409; doi:10.15698/mic2018.02.615)
Supplement: Supplementary file 1 [file mic-05-104-s01.pdf]

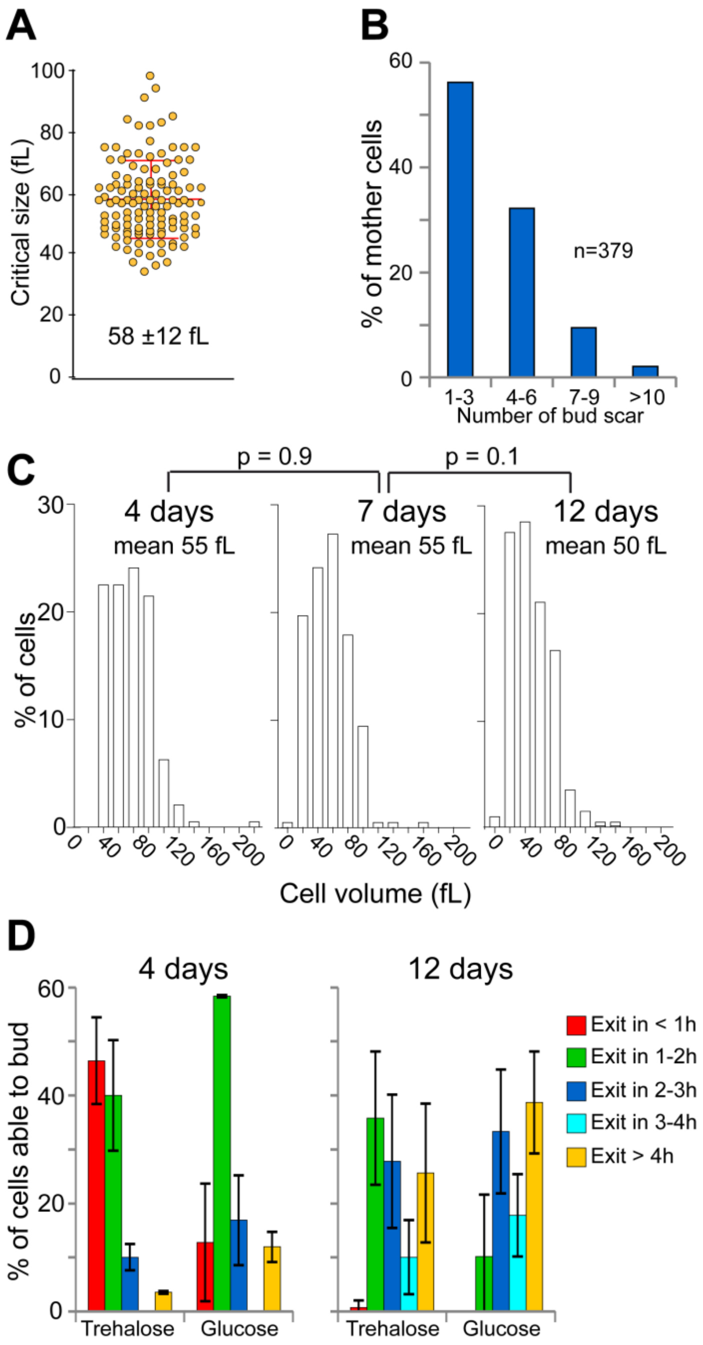

Supplemental FIGURE 1: (A) Quiescence exit critical volume. Mean cell volume at which daughter cells emit a bud upon quiescence exit, irrespectively of the time after refeeding on a YPD-containing microscope agarose pad (n>120, N=2). **(B)** Distribution of the percentage of mother cells accordingly to their replicative age (bud scars number). **(C)** Cell volume distribution in function of cell chronological age *i.e.* the time spend in liquid YPDA culture at 30°C (n>120). P-values and means are indicated. **(D)** Trehalose influences quiescence exit efficiency. Wild type prototroph CEN-PK cells were grown in YP pH 5 containing 4% glucose or 2% glucose + 2% trehalose for the indicated time. Quiescence exit was triggered on a YPD-containing microscope agarose pad and cells were imaged every hour (for each time point n>100 N=3).
